# Coated Zein Polymeric Nanoparticles Loaded with Amlodipine as a Repurposed Antibacterial Ocular Cure for MRSA-Induced Infection: Optimization, In Vitro, Ex Vivo, and In Vivo Assessments

**DOI:** 10.3390/pharmaceutics17101314

**Published:** 2025-10-10

**Authors:** Alaa S. Eita, Amna M. A. Makky, Asem Anter, Islam A. Khalil

**Affiliations:** 1 Department of Pharmaceutics, College of Pharmaceutical Sciences and Drug Manufacturing, Misr University for Science and Technology (MUST), P.O. Box 77, Giza 12566, Egypt; 2 Department of Pharmaceutics and Industrial Pharmacy, Faculty of Pharmacy, Cairo University, El-Kasr El-Aini Street, Cairo 11562, Egypt; 3 Microbiology Unit, Drug Factory, College of Pharmaceutical Sciences and Drug Manufacturing, Misr University for Science and Technology (MUST), P.O. Box 77, Giza 12566, Egypt

**Keywords:** amlodipine, zein, alginate, eyedrop, repurposing, MRSA infection, ocular

## Abstract

**Background:** Amlodipine besylate (AML) is recognized as a calcium channel blocker curative for hypertension. However, the drug emerged recently as an antibacterial cure that competently prevails over resistant strains. **Methods:** Incorporating amlodipine into zein nanoparticles was employed to innovate a suitable carrier for loading and targeting deep corneal infection. The Box–Behnken design was adopted to produce various formulations of amlodipine-loaded zein nanoparticles (AML-ZNs) with diversity in composition concentration (% *w*/*v*), comprising zein, Labrafac, and poloxamer 407. **Results:** Relying on the optimization criterion, the chosen preference formulation concentration (% *w*/*v*) consists of 2.068 for zein, 0.75 for Labrafac, and 1.0 for Poloxamer. Morphological micrography of AML-ZNs showed regular spherical particles in the nanometric scale, and physicochemical characterization procedures confirmed system suitability. While tracking eyedrop optimum features, sodium alginate was selected for coating nanoparticles to improve stability and system viscosity. Both pH and sterility were also considered and maintained. Comparative studies were conducted pre- and post-coating, and the assessed features for the final selected formulation were 349.9 ± 5.8 nm, 0.2186 ± 0.0271, −55.45 ± 1.84 mV, 81.293 ± 0.9%, and 19.3 ± 0.19 cp for size, PDI, surface charge, entrapment, and viscosity, respectively. The AML-ZNs-Alg formulation demonstrates a more controlled pattern of release of roughly 40% of the drug released after 48 h, while the permeation profile shows 37 ± 3.52% permeated after 24 h, confirmed visually. In vitro microbial assay alongside the corneal in vivo microbial and histological pathology evaluation proved the efficacy of amlodipine as an antibacterial agent. **Conclusions:** These findings highlighted that the prepared AML-ZNs-Alg eyedrop can be a promising system as an antibacterial therapy.

## 1. Introduction

Bacterial ocular infections, predominantly keratitis, conjunctivitis, blepharitis, and endophthalmitis, are deemed a vital health concern globally owing to their potential to induce visual impairment [1]. Many causative pathogens can be implicated, while *Staphylococcus aureus* is considered the prevailing etiological agent [2]. The evolution of methicillin-resistant *Staphylococcus aureus* (MRSA) has established more clinical complications and therapeutic challenges. Each of the two strains, healthcare-associated (HA-MRSA) and community-associated (CA-MRSA), has been recognized in ophthalmic infections, considering that the latter is more aggressive with higher resistance [3]. Previous statistics reports stated that MRSA can contribute to 30–40% of serious ocular *S. aureus* infections [4]. The elevated prevalence of resistant strains in ocular infections is considered the foremost challenge for antibacterial therapy, especially fluoroquinolones, the chief line of ocular treatment [5]. Consequently, the emergence of antimicrobial resistance (AMR) to traditional antibiotics necessitates ongoing research to identify prospective innovative methods or discover novel chemical entities that can prevail over the resistant strains [6].

The process of identifying a novel chemical entity that exhibits antibacterial properties is assumed to be intricate, with an extended timeframe and substantial financial burden [7]. The drug repositioning (repurposing) approach employs a previously known approved drug beyond its specific medical scope to overcome the intensive process of developing a novel drug for a particular issue [8]. Hence, lately, the management of bacterial resistance relies on drug repositioning to recognize alternative drugs with recently known antibacterial activity [9]. Various therapeutic drug classes were reported to have efficient antimicrobial activity against resistant strains, including MRSA: for instance, NSAIDs, antirheumatic antidepressants, antiparasitics, and antidiabetics [10]. Furthermore, as Hua et al. also noticed in a previous study, the antihypertensive drug amlodipine exhibits an antibacterial property [11].

The dihydropyridine calcium channel blocker amlodipine (AML) is a recognized therapeutic agent in treating cardiovascular disorders by suppressing calcium influx, leading to the dilatation of blood vessels’ smooth muscle [12]. On the other hand, Kumar et al.’s previous study proved AML’s antibacterial activity upon screening for drug efficiency over an assortment of Gram-negative and Gram-positive bacterial species [13]. Moreover, Sharma et al., upon evaluating AML’s antimicrobial activity, established the potential activity of AML in diminishing distinct virulence traits, comprising the establishment of microbial biofilm and resistance to oxidative stress. The AML mechanism that interprets antibacterial activity relies on bacterial cell membrane disruption, which triggers the liberation of biological components involving proteins and nucleic acids. Accordingly, cell death emerges in response to disrupting cell membrane integrity [10]. Furthermore, AML’s capability to suppress beta-lactamase contributes to overcoming the drug resistance pathway [14]. Hence, employing AML in the repurposing field as an antibacterial cure can eliminate the resistance challenge of traditional antibiotics.

Various ophthalmic drug delivery obstacles, comprising the epithelial layer of the cornea and blood aqueous barriers, tear turnover, and nasolacrimal drainage, decrease drug contact time and bioavailability for topical therapy [15]. Using AML as a topical antibacterial remedy for ocular infection necessitated reformulation employing nanotechnology to overcome the drawbacks of traditional treatment and enhance efficacy [16]. Accordingly, polymeric nanoparticles were established as prospective carriers for ophthalmic drug delivery based on their efficiency for elevated drug stability, extended precorneal contact time, maintaining drug depot action, reinforcing drug penetration, and enhancing bioavailability [17]. Zein, a corn protein, is considered to have a slight amphiphilic nature but prefers the hydrophobic amino acids. Zein has been highlighted recently for its safety, biodegradability, biocompatibility, and ability to self-structure into spherical particles in the nanometric size and load both lipophilic and hydrophilic drugs [16]. Zein polymeric nanoparticles were applied as promising carriers for a broad therapeutic scope, incorporating various agents such as resveratrol, artemether, and curcumin. However, Zein’s limited stability and capability to aggregate in response to changing physiological conditions hinder its distinct features as a delivery system. Hence, the previous studies emphasize that further stabilization techniques, such as coating or the addition of stabilizer agents, are indispensable [18,19].

Studies have extensively adopted the coating approach in enhancing zein stability, encompassing lecithin and various polymers such as alginate, chitosan, and sodium caseinate [20,21,22,23]. Sodium alginate, an anionic polymer, is widely used in coating nanoparticles to uphold system stability and prevent agglomeration [24,25]. Accordingly, previous researchers nominated alginate for coating zein nanoparticles owing to its mucoadhesiveness, biocompatibility, and biodegradability features [21,22]. Alginate yields an additional advantage to eyedrop preparation upon adjusting viscosity for more adaptation with lacrimal drainage [26]. However, extra features regarding eyedrop formulation should be considered to achieve the most appropriate system, including stability, sterility, pH, and isotonicity. Tracking these properties ensures patient comfort and improves therapeutic performance [27].

This research intends to illuminate the AML antibacterial activity upon integrating the drug into polymeric zein nanoparticles (AML-ZNs) to defeat ocular barriers and modify drug release and penetration. In addition, alginate coating was implemented on the AML-ZN surface to uphold zein system stability and adjust viscosity to ensure system competence as eyedrops. A Box–Behnken design was employed to systematically evaluate and optimize AML-ZN formulations to determine the most desirable concentration for ZN composition. Numerous features for each ZN and ZN-Alg were assessed in addition to comparative studies before and after coating to confirm the preference of coating and to validate overall suitability and stability of the system. The eyedrops’ crucial attributes, encompassing viscosity, pH, and preservatives, were considered to ensure system adequacy. Moreover, in vitro microbiological and in vivo assessments were executed to ensure AML efficiency as an antibacterial cure.

## 2. Materials and Methods

### 2.1. Materials

The EVA Pharma company in Egypt kindly gifted amlodipine besylate. Labrafac was obtained from Gattefosse, Saint-Priest, France. Zein protein (M.wt 22–27 KDa) was received from Acros Organics Co., Geel, Belgium. Poloxamer 407, Sodium Alginate, Polyethylene glycol 400, and Cellulose membrane (NMWCO:12000) were bought from Sigma-Aldrich (St. Louis, MO, USA). Propylene glycol, methanol, and ethanol were purchased from El-Nasr Pharmaceutical Co. (Cairo, Egypt). All other chemicals involved in the whole study were of high-purity grade.

### 2.2. Zein Polymeric Nanoparticles

#### 2.2.1. Formulation of Amlodipine-Loaded Zein Nanoparticles

Amlodipine-loaded zein nanoparticles (AML-ZNs) were prepared using an antisolvent precipitation technique as previously described with slight modification [28]. A 50 mg dose of amlodipine was solubilized in 1 mL of methanol; 1% (*w*/*v*) PEG 400 was added as a stabilizer, in addition to Labrafac and Poloxamer 407, utilizing variable amounts according to the employed design. Zein was mixed with 2 mL of 90% *v*/*v* ethanol at room temperature until complete solubilization. The zein solution was added dropwise to the previously prepared solution containing AML, with uninterrupted stirring at 1500 rpm using a magnetic stirrer for 15 min to confirm complete solubilization. The mixture was injected dropwise into deionized water (pH 5–5.5), fourfold the volume of the alcoholic solution, maintaining constant stirring at 1500 rpm overnight to ensure complete evaporation of the alcoholic mixture, leading to nanoparticle formation. The resulting formulation was stored overnight at 4 °C for further assessment.

#### 2.2.2. Experimental Design of AML-Loaded Zein Nanoparticles

The response surface methodology (RSM) provides minimal experimental trials for optimal outcomes. All RSM designs are proficient in recognizing the main effects and interactions among all factors in constructing the mathematical models [29]. This study applied a Box–Behnken design for (AML-ZN) formulations using Design-Expert^®^ software (version 13, Stat-Ease Inc., Minneapolis, MN, USA) to assess different concentrations of investigated factors. The manipulated variables were zein concentration (%, X_1_), Labrafac concentration (%, X_2_), and Poloxamer 407 concentration (%, X_3_), while particle size (nm, Y_1_), polydispersity index (Y_2_), zeta potential (mV, Y_3_), and entrapment efficiency (%, Y_4_) were considered measured outcomes, as represented in Table 1. Determined by the amalgamation of different variables’ levels, 17 experimental runs were constructed by the software. Optimization suggestions called for specific variable selections that contribute to the most acceptable formulation with minimal particle size and polydispersity index measurements, as well as maximum zeta potential and entrapment efficiency values.

#### 2.2.3. Assessment of Amlodipine-Loaded Zein

##### Dynamic Light Scattering Analysis

The dynamic light scattering (DLS) technique was employed at 25 °C, utilizing Zetasizer (Malvern Instruments, Malvern, UK) for measuring zeta potential (ZP), polydispersity index (PDI), and particle size (PS). AML-ZN dispersion was assessed after 10-fold dilution using deionized water; thereafter, the mean and standard deviation were computed in triplicate [30].

##### Evaluation of Entrapment Efficiency

The amount of drug entrapped (EE) in the ZN system was measured directly by centrifuging 1 mL of the prepared formulation using a cooling centrifuge (Sigma 3 K 30, Sigma Laborzentrifugen GmbH, Osterode am Harz, Germany) at 2000 rpm for 2 h at 4 °C. The supernatant was removed, and the pellet was lysed using a mixture of methanol: ethanol (1:1) until a clear solution was obtained. The clear solution was analyzed using a UV–Vis spectrophotometer at 361.5 nm (media of methanol/ethanol) (Shimadzu UV-1650, Shimadzu Corporation, Kyoto, Japan) [23]. EE% was quantified in triplicate using the following equation:

(1) $$EE\%=\frac{E_{AML}}{T_{AML}}\times 100$$

T_AML_ and E_AML_ denote the total amlodipine and entrapped amlodipine in the ZN system.

#### 2.2.4. Specification of Optimized AML-ZN Formula

##### Morphological Micrography

The morphological features of AML-ZNs were depicted with a transmission electron microscope (TEM; CM12; Philips, Andover, MA, USA). The optimized formulation from the BBD trials was inspected to visualize the system structure. A proper sample dilution was stained negatively with an aqueous solution of 2% phosphotungstic acid stain and left to dry on a carbon grid before being analyzed at 80 kV using TEM [31].

##### Differential Scanning Calorimetry

Thermal examination was implemented to display the thermograms of pure AML, zein, Poloxamer 407, Labrafac, PEG 400, and AML-ZNs using a differential scanning calorimeter (DSC, Shimadzu TA-60, Japan). The DSC instrument was calibrated using indium as a reference material to confirm enthalpy measurements. A constant nitrogen purge was maintained at a 50 mL/min flow rate to deliver an inert atmosphere and prevent sample oxidation. An amount equivalent to 5 mg of each sample was enclosed in an aluminum pan and scanned at a heat range from 25 to 250 °C at a scanning rate of 10 °C/min utilizing nitrogen gas as a purge [32].

##### Fourier Transform Infrared Spectroscopy

The IR spectra of AML, zein, Poloxamer 407, Labrafac, PEG 400, and AML-ZNs were analyzed using Fourier transform infrared spectroscopy (FT-IR) (Shimadzu IR-Affinity-1, Japan) to observe any AML-excipient incompatibility. Roughly 3 mg of each sample, post-mixing with KBr, was compacted into a disk. Scanning was applied at ambient temperature in the 400–4000 cm^−1^ range with a resolution of 4 cm^−1^ and speed of 2 mm/s [33].

### 2.3. Incorporation of Nanoparticles in Eyedrops

The AML-ZNs were incorporated into an optimum eyedrop formulation after tracking specific features to designate the most suitable system. Rapid ocular drainage and the tear-limited ability to alter pH required further assessment to maintain appropriate viscosity and pH [34]. In addition, preservatives are in demand in ophthalmic formulations to ensure sterility of multi-dose preparations [35].

#### 2.3.1. Viscosity

The viscosity of the eyedrops establishes a critical role in retention time, patient comfort, and effectiveness [36]. Using the Brookfield DV3T Viscometer (Brookfield Engineering Laboratories, Inc., Middleboro, MA, USA), viscosity was measured in triplicate at 25 °C ± 2 °C and 250 rpm using spindle 40 [37].

Alginate is widely used in pharmaceuticals as a thickening agent, stabilizer, emulsifier, chelating agent, and encapsulation, swelling, and suspending agent. The negatively charged state of alginate is favorable for the coating layer and can be used to improve viscosity. However, including a particular bivalent cation (crosslinker) in the alginate solution leads to gel formation with higher viscosity through ion exchange [38].

Variable amounts of sodium alginate and propylene glycol (PG) as a plasticizer were dissolved in deionized water under stirring at ambient temperature. The AML-ZN formulation was injected dropwise into the alginate solution and left for 2 h on a magnetic stirrer at room temperature to ensure efficient coating as per the previously reported method with slight modification [25]. The produced formulations were stored overnight at 4 °C for further evaluation and comparative study. The viscosity of all prepared formulations was measured alongside AML-ZNs’ characteristic features to identify the optimum formulation, considering the desired viscosity limit for optimum eyedrop flow and residence time.

##### Dynamic Light Scattering

The measured parameters, zeta potential (ZP), polydispersity index (PDI), and particle size (PS), were reassessed post-coating for all formulations using the dynamic light scattering (DLS) technique as previously described.

##### Entrapment Efficiency

The amount of drug entrapped (EE) in all AML-ZNs-Alg formulations was assessed directly, as previously mentioned in Section Evaluation of Entrapment Efficiency, and indirectly to prove results by calculating the unentrapped amount after centrifuging 1 mL of the prepared formulation using a cooling centrifuge (Sigma 3 K 30, Germany) at 2000 rpm for 2 h at 4 °C. The supernatant was collected and analyzed using a UV–Vis spectrophotometer at 361.5 nm (Shimadzu UV 1650 Spectrophotometer, Japan). EE% was quantified in triplicate using the following equation:

(2) $$EE\%=\frac{T_{AML}-U_{AML}}{T_{AML}}\times 100$$

T_AML_ and E_AML_ symbolize the ZN system’s total amlodipine and unentrapped amlodipine.

##### Vesicle Morphology

TEM was utilized to assess the morphological features of the AML-ZNs-Alg coating system to detect changes in morphological features and ensure efficient coating. The selected optimum formulation from the alginate coating study was inspected to visualize the system structure using the previously described technique.

#### 2.3.2. pH

The pH values were measured in triplicate for the prepared AML-ZN eyedrop formulations. The pH value was estimated at room temperature using a calibrated pH meter (Metrohm; Nordic ApS, Herisau, Switzerland). The pH of an ocular formulation may be buffered to promote patient comfort and boost the drug stability and bioavailability [39]. The ideal pH should be compatible with tears, a neutral value of about 7.4 [15]. The AML-ZNs-Alg final formulation pH was adjusted to 7.4 using phosphate buffer solution.

#### 2.3.3. Preservatives

Preservatives are considered one of the crucial additives to ophthalmic formulations. The prevention of microbial growth or any undesirable reactions is essential due to the eye’s higher sensitivity [35]. Sodium benzoate is widely utilized as a preservative in foods, cosmetics, and pharmaceutical formulations, and it was the choice of this study due to its antibacterial and antifungal effects [40]. The conventional concentration of sodium benzoate in liquid pharmaceutical preparations ranged from 0.1% to 0.2% *w*/*w* [41]. Accordingly, 0.1% sodium benzoate was solubilized in the final optimized AML-ZNs-Alg eyedrop formulation, and the final formulation after pH adjustment and preservative addition was adopted for further comparative studies with the optimized AML-ZN formulation to prove validation.

### 2.4. Comparative Studies of AML-ZNs and AML-ZNs-Alg Eyedrops 

#### 2.4.1. Dynamic Light Scattering Analysis and Entrapment Efficiency

The final preparation after AML-ZNs were incorporated into the alginate coat and post-optimization of all eyedrop features were re-measured in terms of PS, PDI, ZP, and EE to investigate the applicability and suitability of the optimized AML-ZNs-Alg eyedrops.

#### 2.4.2. In Vitro Release Study

A modified USP dissolution tester apparatus type II (Hanson Research Corp, Chatsworth, CA, USA) was implemented in the comparative study of the drug release profile of the AML-ZNs-Alg system, compared to AML suspension and AML-ZNs. A modified USP dissolution tester was utilized to simulate the Franz diffusion cell with applicability for a higher volume. An adequate volume of the evaluated samples, equivalent to 8 mg AML, was inserted into two side-opened tubes with a surface area equal to 5 cm^2^, with one end sealed with a cellulose membrane and the other fastened to the shaft of the dissolution device [42]. The release medium was 100 mL of PBS (pH 7.4) containing 10% ethanol, kept under an adjusted temperature of 37 ± 0.5 °C with uninterrupted mixing at 100 rpm [43]. As noticed by Kapoor et al., ethanol can be added to the AML release media to retain the sink conditions [44]. At predetermined time intervals, 2 mL aliquots of the samples from the release medium were withdrawn and replaced with fresh medium. Samples were assessed at each time interval utilizing a UV–Vis spectrophotometer at 364.5 nm (medium of PBS/10% ethanol) to calculate the released amount of AML, considering the correction of the dilution effect during aliquot withdrawal as represented in the following equation:

(3) $$Released\;\%\;\left(corrected\right)=\left(\frac{{Cn}_{AML}\cdot V+\sum_{p=1}^{n-1}{Cp}_{AML}\cdot \nu \;}{T_{AML}}\right)\times 100$$

As symbols indicate, Cn_AML_: Concentration of drug in the measured sample, Cp_AML_: Concentration of the drug in the previous samples, V: Total volume of the dissolution medium, ν: Volume of each aliquot withdrawn, T_AML_: Total amount of AML in the study.

Data derived from the release profile were kinetically interpreted by adopting different models, such as zero order, first order, Hixson–Crowell, Korsmeyer–Peppas, and Higuchi models, looking for the ideal fitting model with a superior correlation coefficient (R^2^) using DDsolver software (version 1.0, 2010). Data interpretation was based on the clarification of Costa and Sousa Lobo and Zhang et al. [45,46].

#### 2.4.3. Ex Vivo Permeation and Deposition Studies

##### Corneal Permeation

The ex vivo comparative assessment of AML from ZNs and ZNs-Alg systems to track permeation through corneal tissue was conducted using the modified USP dissolution tester apparatus type II, utilizing cow cornea for permeation experiments. The fresh cornea was fixed on one end of two side-opened tubes, and the other end was affixed to the shaft of the device. AML-ZNs, AML-ZNs-Alg system (equivalent to 8 mg), and 8 mg of drug suspension were placed inside the tube. Additionally, the permeation study medium consisted of 100 mL PBS (pH 7.4) containing 20% ethanol, kept at 37 ± 0.5 °C under constant mixing at 100 rpm for 24 h [47]. Two-milliliter aliquots were withdrawn from the medium at planned timepoints and substituted immediately with fresh medium. The collected samples were filtered (0.45 µm membrane) and analyzed employing a validated HPLC method. The cumulative amount of AML permeated from the investigated formulations was displayed versus the time used to evaluate the permeation rate. The permeation flux (Jss) parameter was also calculated.

##### Corneal Deposition

After the execution of the permeation study, the corneal tissues were delicately removed, washed, and homogenized with a predetermined volume of methanol. The corneal tissues were kept in methanol overnight and then sonicated for 30 min in a bath sonicator (Elmasonic S 60 H, Elma, Bangkok, Thailand) to ensure adequate extraction of the drug deposited in the corneal epithelium. The supernatant was separated and filtered using a 0.45 µm membrane, and then analyzed utilizing a validated HPLC [48].

##### HPLC Assay

For AML quantitation in ex vivo studies, an earlier-stated and validated HPLC method established by Sankar et al. was applied with slight modifications [49]. The HPLC (Agilent Technologies, Santa Clara, CA, USA) with autosampler was employed, and the UV detector was set at λ _max_ 364.4 nm. The mobile phase comprised buffer solution (pH 3) and acetonitrile (50:50, *v*/*v*) with a 1 mL/min flow rate at 40 °C. Before injection, all samples were filtered with a 0.45 μm Millipore filter to prevent column blockage. A volume of 50 μL from each sample was injected into the analytical column. The analytical HPLC method was validated in terms of linearity, specificity, precision, limit of detection, and recovery.

#### 2.4.4. Ex Vivo Visualization Study

In the ex vivo visualization study, a fluorescent dye was loaded into the ZNs and ZNs-Alg systems instead of AML to observe, track, and compare the penetration capability of both systems in corneal layers using confocal laser scanning microscopy (CLSM).

##### Preparation of FDA-Loaded Formulations and Experiment Setup

Fluorescein Diacetate (FDA) dye was loaded instead of AML in ZNs and ZNs-Alg systems. The permeation study was performed for FDA-ZNs and FDA-ZNs-Alg for 8 h, similarly to the whole condition of the conducted ex vivo corneal permeation study. Subsequently, the corneal tissue was collected and washed with normal saline to remove any excess dye, and then it was retained at −20 °C until CLSM visualization.

##### Confocal Laser Scanning Microscopy Study

A small longitudinal section of the corneal tissue was inspected using a microscope (LSM 710; Carl Zeiss, Oberkochen, Germany). The FDA dye excitation wavelength was 488 nm, and the emission wavelength was 530 nm. Considering corneal layers, the observation yielded qualitative and quantitative data regarding the evaluated formulations. Optical scanning was conducted across a depth range of 0 µm to 150 µm, utilizing increments of 10 µm. The penetration attributes of the ZNs and ZNs-Alg systems within the corneal tissue were elucidated by fabricating a three-dimensional graphical illustration implementing a Z-stack model [50].

### 2.5. Influence of Storage on AML-ZNs-Alg Eyedrops

The optimum AML-ZNs-Alg formulation was stored at 4 ± 1 °C, in tightly sealed, amber-glass vials for 90 days to ascertain system stability during storage. PS, PDI, ZP, EE, viscosity, pH, and physical appearance were tracked to detect any agglomeration or changes in the formulation’s physicochemical stability. Measurements were taken for a freshly prepared sample, estimated as time zero, in addition to samples investigated at 30, 60, and 90 days. The evaluation was conducted in triplicate for all samples to ensure accurate results.

### 2.6. In Vitro Antimicrobial Assessments

#### 2.6.1. Microbial Test Cultures

The adopted standard strains employed in this work, *Staphylococcus aureus* and Methicillin-resistant *Staphylococcus aureus* (MRSA), were procured from the culture collection unit at the regional Center for Mycology and Biotechnology in Al Azhar University. Bacterial strains were conserved at ultra-low frozen storage (–80 °C) in 15% glycerol stocks. BHI broth was utilized to grow one colony of *S. aureus* and MRSA. Subsequently, the bacteria were centrifuged for 10 min at 10,000× *g*, washed, and resuspended in sterile phosphate-buffered saline (PBS). The bacterial suspension was set at a final density of 2.6 × 10^5^ colony-forming units CFU/mL. Isolated strains were preserved at 37 °C on mannitol salt agar (MSA). Strain purity was confirmed by detecting the morphology of the cell and colony, and applying biochemical tests (catalase and coagulase) relying on standard procedures.

#### 2.6.2. Agar Well Diffusion Method

The antibacterial assay was conducted utilizing the Bauer technique, referring to the Clinical Laboratory Standard Institutions guidelines for agar well diffusion [51,52]. A 100 μL volume comprising 1.5 × 10^8^ colony-forming units/mL of the adopted strain’s suspension was evenly swabbed on an agar plate loaded with 25 mL of sterile Muller–Hinton Agar (MHA). The inocula were prepared from the overnight incubated cultures employing the antimicrobial assay’s direct colony method. Discrete colonies were gathered by sterile wire loop and suspended in sterile 0.85% saline; the turbidity of the suspension was aligned with the 0.5 McFarland standard. Using a sterile cork borer, a well of 5 mm diameter was produced on the seeded surface, and a proper dilution volume (50 μL) of the assessed formulations (4 mg/mL) was deposited into the wells. The commercially available Optiflox^®^ eyedrop was provided as a commercial control. The culture plates were maintained at 37 °C for 24 h, and the inhibition aspects were assessed manually using a standard scale measuring the apparent inhibited zone around the well. The study was carried out in triplicate to confirm the outcomes statistically.

#### 2.6.3. Micro-Dilution Assay for MIC Estimation

The broth dilution method, under the recommendation of the Clinical and Laboratory Standards Institute (CLSI) guideline, was employed to determine MIC concentration [53]. Successive two-times dilutions of all investigated formulations were prepared in sterile-capped universal bottles. A 2 mL volume of the overnight cultured strains was supplemented with 2 mL of each investigated sample dilution, followed by vortexing and 18 h of culturing at 37 °C. A prepared broth inoculated with the studied strains was used as a control to determine inhibition. The commercially available Optiflox^®^ was used as a commercial control. Consequently, based on the growth turbidity, the MIC values were identified as the lowest concentration of each formulation inhibiting the growth of the adopted strains [54,55].

### 2.7. In Vivo Assessments

The in vivo investigations were carried out on healthy albino male rabbits weighing 2–2.5 Kg, where each rabbit was housed in a separate cage, fed a standard dry meal, and water ad libitum under standard conditions of temperature (25 °C) and humidity (50–60%). This study was executed per the regulations confirmed by the Department of Pharmacy’s ethical committee, Cairo University (protocol serial number PI 3905).

#### 2.7.1. Evaluation of Ocular Irritation

The ocular Draize test was conducted to ensure the safety of the developed formulation. A 100 μL volume of the evaluated formulation was loaded in the rabbit’s left eye conjunctival sac thrice daily for 14 days, while the right eye served as a control. The rabbit eye was examined for inflammation, ocular hypersensitivity, or hyperlacrimation. A histopathological study was performed using the method described in the Section 2.7.3 to confirm formulation compatibility and exclude ocular damage.

#### 2.7.2. In Vivo Antimicrobial Evaluation

All rabbits were anesthetized for the induction of MRSA keratitis, followed by ocular washing. A 100 μL volume of 2.6 × 10^5^ CFU/mL of MRSA strain was injected into the corneal stroma. The rabbits’ eyes were monitored daily until signs of MRSA keratitis occurred. A parallel design of four groups, each having two randomly selected rabbits (total = 8 rabbits, n = 2), was implemented. Group I was assigned to the infected eye with no treatment (positive control). Group II was treated with a dose of the commercial antibacterial drug (Optiflox^®^) 4 times daily. Group III was assigned to the AML-ZNs-Alg eyedrops with one dose every 24 h. Group IV was assigned to the blank ZNs-Alg formulation. The allocated therapies were applied daily, and treatment was continued for 7 days after the induction of bacterial keratitis. The conjunctival congestion, redness, mucopurulent discharge, chemosis, corneal infiltrate, and corneal edema were evaluated as indicators of bacterial keratitis. After 7 days of treatment, rabbits were humanely sacrificed by decapitation using phenobarbital sodium injection for anesthesia in the marginal vein. Eye specimens were gathered from the investigated groups, considering the left eye for microbiological and the right eye for histopathological assessment. For the microbial assessment, homogenized corneal samples were cultured in mannitol salt agar (MSA) for 24 h at 37 °C, followed by colony-forming unit (CFU) enumeration [56].

#### 2.7.3. Histopathological Dissection

The histopathological analysis was performed on the whole rabbit eye, which was washed with normal saline, fixed in 10% buffered formalin for 24 h, rinsed, dehydrated, trimmed, and embedded in paraffin. Tissue specimens of 5 µm-thick sections were affixed to glass slides [50]. The acquired tissue sections were deparaffinized using xylol and stained using hematoxylin and eosin (H&E) for histopathological evaluation through the electric light microscope [57].

### 2.8. Statistical Analysis of Data 

Students’ *t*-test and one-way and two-way analyses of variance (ANOVA), accompanied by Tukey’s post hoc tests, were adopted to verify statistically significant differences among the investigated samples. A significance level was developed at 0.05, and (* *p* < 0.05, ** *p* < 0.01, *** *p* < 0.001, and **** *p* < 0.0001) was declared to exhibit statistical significance. Data analysis performed using GraphPad Prism software version 6.07 (GraphPad Software Inc., San Diego, CA, USA).

## 3. Results and Discussion

### 3.1. Experimental Analysis of AML-ZN Formulation

The selection of the most appropriate concentrations of zein in nanoparticle formulation is crucial, as they affect system stability, EE, PS, and the release from the formulated ZNs [58]. Higher zein concentration affects drug loading positively; otherwise, it can enhance the size of nanoparticles [59]. Elevated nanoparticle size requires sufficient stabilizing agents to maintain stability. At the same time, higher increases in zein concentration (2.5–15%) resulted in a greater augmentation in PS, which can cause aggregation [28]. As reported by Podaralla and Perumal, enhanced surfactant concentrations (>0.45% (*w*/*v*)) can result in smaller PS and uphold ZNs’ stability [60]. In addition, Labrafac, as a lipid carrier, can improve lipophilic drug solubility and facilitate the surfactant (Pluronic) stabilizing mechanism. Moreover, Labrafac can assist in controlling drug release from the zein matrix and contribute to sustained release of the drug [61]. Therefore, the BBD 17 experimental trials (Table 2) were designed to study the effect of zein, Labrafac, and Poloxamer concentrations on the evaluated responses. The experimental trials’ data analysis was driven by Design-Expert® software, version 13 (Stat-Ease Inc., Minneapolis, MN, USA) using analysis of variance (ANOVA). The ANOVA findings reflect statistically significant outputs revealing different models, as demonstrated in Table 3.

#### 3.1.1. Impact of the Variables on Evaluated Responses

##### Particle Size

AML-ZN particle size results ranged from 173.7 ± 9.69 nm to 237.4 ± 3.87 nm, as represented in Table 3, indicating the nanometric range for all prepared ZN formulations. The model fits a linear model with a nonsignificant lack of fit (*p* = 0.9571). The following equation shows the model for PS (Y_1_):

PS = +210.41 + 19.44X_1_ + 10.73X_2_ − 10.61X_3_ (4)

**Figure 1:**
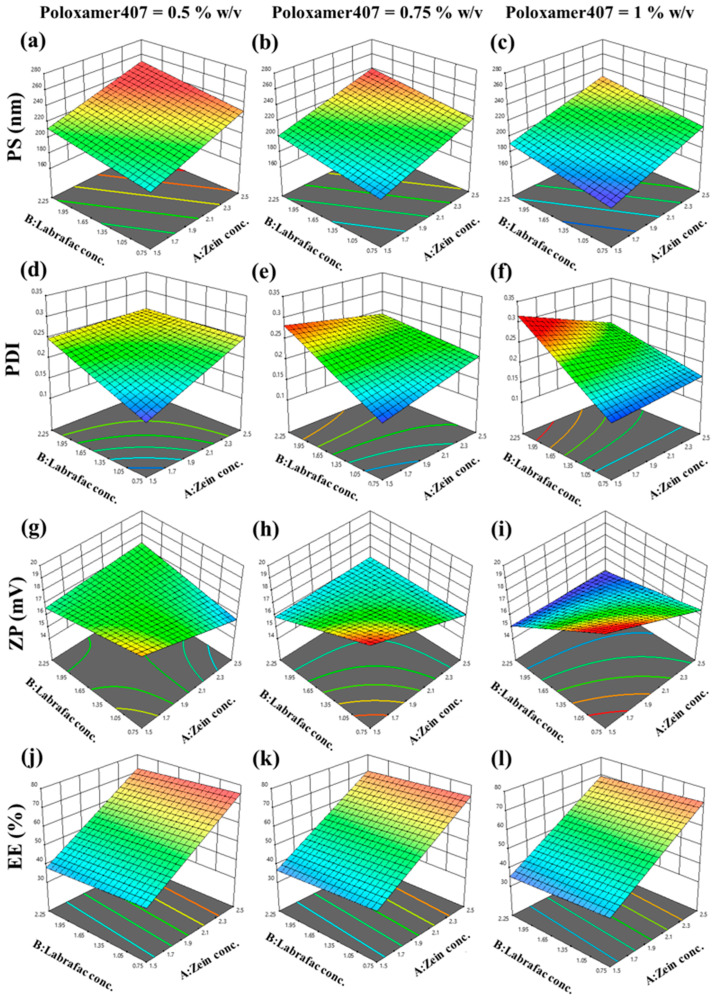
Three-dimensional plots of BBD upon the evaluation of particle size (PS) at the different levels of Poloxamer407 (**a**) 0.5% (*w*/*v*), (**b**) 0.75% (*w*/*v*), and (**c**) 1% (*w*/*v*), polydispersity index (PDI) at the different levels of Poloxamer407 (**d**) 0.5% (*w*/*v*), (**e**) 0.75% (*w*/*v*), and (**f**) 1% (*w*/*v*), zeta potential (ZP) at the different levels of Poloxamer407 (**g**) 0.5% (*w*/*v*), (**h**) 0.75% (*w*/*v*), and (**i**) 1% (*w*/*v*), and entrapment efficiency (EE) at the different levels of Poloxamer407 (**j**) 0.5% (*w*/*v*), (**k**) 0.75% (*w*/*v*), and (**l**) 1% (*w*/*v*) considering the different levels of other variables; results represent the significant impact of various variable ratios on all responses.

As depicted in Figure 1a–c, an increase in zein concentration level (X_1_) positively impacted particle size (*p* < 0.0001), which can be explained by enhanced dispersion system viscosity with elevated zein concentrations. Increasing viscosity forms a barrier to the antisolvent precipitation mechanism, affecting the nucleation step’s efficiency in producing small particles [60]. Labrafac concentration (X_2_) positively influenced zein nanoparticle size (*p* = 0.0006), as a lipid vehicle entrapped in the nanoparticle matrix led to elevated core volume and enhanced particle size [62]. Lipid coexistence in nanoparticle matrix also increases viscosity, which may amplify aggregation by inhibiting nucleation [63]. Additionally, the hydrophobic nature of Labrafac may augment hydrophobic interaction with zein, contributing to particle fusion [64]. Otherwise, a negative relation with PS was disclosed regarding the effect of poloxamer concentration (X_3_) (*p* = 0.0006). Poloxamer 407 is an amphiphilic triblock copolymer that comprises polyethylene oxide (PEO) and polypropylene oxide (PPO) units, which add to its hydrophilic–lipophilic balance (HLB) [65]. At a concentration of 1% as reported by Elmowafy et al., the poloxamer hydrophobic block can be adsorbed entirely on the surface of the hydrophobic drug, and a steric barrier resulting from the PEO chain avoids aggregation and upholds size diminishment and stability [66].

##### Polydispersity Index

PDI is a crucial factor that substantiates uniformity or diversity of nanoparticle size distribution. It was established that the monodispersed system should acquire PDI values between 0.1 and 0.5 [67]. All polydispersity index data points for AML-ZN formulations proved uniformity, ranging from 0.145 ± 0.0053 to 0.284 ± 0.0142, as elucidated in Table 4. The model fits a 2FI model with a nonsignificant lack of fit (*p* = 0.9537), and the following equation stands for the PDI (Y_2_) model:

PDI = 0.2198 + 0.0053X_1_ + 0.0422X_2_ − 0.0041X_3_ − 0.0255X_1_X_2_ − 0.0229X_1_X_3_ + 0.0149X_2_X_3_ (5)

Figure 1d–f clarify the positive impact of different Labrafac concentrations (X_2_) on PDI, which is considered significant (*p* < 0.0001). The effect of Labrafac can be explained, as it can be reassembled in a nanoparticle matrix; this enhancement of lipid content in the core can result in a higher particle size with an elevated tendency to aggregate [68,69,70]. As PDI can be an indicator of particle aggregation, it may be enhanced as a response to the clumping of larger particle sizes [71].

As reflected by Equation (5), the interaction of zein concentration with both Labrafac and Poloxamer shows a significantly negative effect on PDI, with *p* = 0.0023 and *p* = 0.0046 for X_1_X_2_ and X_1_X_3_, respectively. The diminution effect of X_1_X_2_ can be explained by the structure of zein, a self-assembly amphiphilic polymer affected by HLB. Oily vehicles can enhance zein particle curvature by potent hydrophobic interaction and develop an even smaller spherical size distribution [72]. Additionally, regarding X_1_X_3_, incorporating surfactants, such as Poloxamer (pluronic F127), in the zein matrix can reinforce uniform size distribution and inhibit aggregation, thereby decreasing PDI [73,74].

##### Zeta Potential

AML-ZN zeta potential responses exhibited a 2FI model with a nonsignificant lack of fit (*p* = 0.8132), and ZP reveal values ranging from 15.19 ± 0.56 mV to 18.87 ± 0.14 mV as presented in Table 3. The equation-defined ZP (Y_3_) model is symbolized as follows:

ZP = 16.83 − 0.6825X_1_ − 0.7700X_2_ − 0.1800X_3_ + 0.8350X_1_X_2_ − 0.2300X_1_X_3_ − 0.8150X_2_X_3_ (6)

ZP indicates nanoparticle surface charge; despite the sign, greater absolute values exhibit higher repulsive forces among particles, which minimize aggregation and maintain system stabilization [75]. Zein significantly influences zeta potential, which is predominantly related to system pH. As previously noted [76], ZP values of zein nanoparticles can vary from highly negative to highly positive charges contingent on the pH, relying on the isoelectric point (pI). The pI of zein is around 5.5–6.2; in this pH range, the electrostatic repulsion was depleted, giving rise to agglomeration [76]. In this study, the pH of dispersed media was around 5–5.5; therefore the zeta potential revealed a positive charge around 20 mV, consistent with Tortorella et al.’s previous study [19]. ZP nearly ±20 mV was mentioned as a convenient value for corneal application [77]. As illustrated in Figure 1g–i, a scant significant decrease in ZP owing to zein concentration (X_1_) (*p* = 0.0310) and Labrafac concentration (X_2_) (*p* = 0.0178) may be associated with their positive influence on PS. Enhancement in nanoparticle size reduces the surface area and diminishes the charged groups responsible for repulsive forces and stability [78].

##### Entrapment Efficiency

Entrapment or encapsulation efficiency is the estimated amount of loaded drug in the nanocarrier matrix. ZNs’ loaded capacity of AML in different formulations fit a linear model with a nonsignificant lack of fit (*p* = 0.6178). Outputs of entrapment evaluation ranged from 33.04 ± 1.65% to 74.04 ± 1.70%, as clarified in Table 3. The model representing EE% (Y_4_) is symbolized in the equation as stated:

EE = 55.53 +17.03X_1_ − 1.71X_2_ − 1.44X_3_ (7)

As defined in Figure 1j–l, the effect of zein concentration (X_1_) is the only factor that significantly increases entrapment efficiency (*p* < 0.0001). Zein is an amphiphilic polymer with a distinctive structure, mainly composed of hydrophobic amino acids, for instance, proline, leucine, and alanine [79]. An elevated entrapment percentage of AML with higher zein concentration might be assigned to the zein hydrophobic matrix that upholds nanoparticle fabrication and lipophilic drug encapsulation, which is desirable for AML entrapment [80].

#### 3.1.2. Optimization of AML-ZNs Using Box–Behnken Design

**Figure 2:**
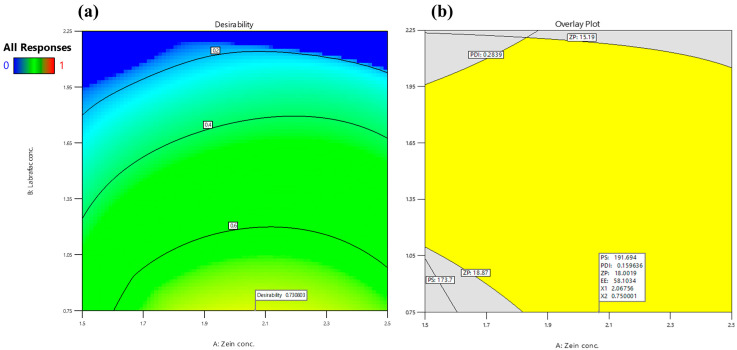
Optimization of BBD design regarding variables and desired evaluated responses; (**a**) Desirability contour plot, and (**b**) Graphical optimization contour plot.

The optimized AML-ZN formulation was adopted according to the previously stated constraints in Table 1. It revealed a maximum desirability of 0.731, as demonstrated in Figure 2a. The optimized selected mixture comprised 2.068% (*w*/*v*) zein concentration (X1), 0.75% (*w*/*v*) Labrafac concentration (X2), and 1% (*w*/*v*) Poloxamer concentration (X3). Consequently, the observed actual responses of the optimized AML-ZNs were 185 ± 4.5 nm, 0.157 ± 0.013, 17.61 ± 0.72 mV, and 59.25 ± 2.72%, showing good correlation with predicted values (Figure 2b), as PS, PDI, ZP, and EE were 191.679 nm, 0.1596, 18 mV, and 58.09%, respectively. The PS and ZP biodistribution curves for optimized AML-ZNs, as demonstrated in Figure S1, revealed a unimodal peak. Regarding bias percentage calculation, all the evaluated actual values for the optimized AML-ZNs showed less than a 5% difference from the predicted values and manifested within the 95% prediction interval, pointing to the statistical design’s validity and affirming its prediction proficiency within data uncertainty.

### 3.2. Specification of Optimized AML-ZN Formula

Further evaluation was conducted on the optimized AML-ZN formulation, which exhibited the highest desirability value and met the intended targeted features.

#### 3.2.1. Morphological Micrography

**Figure 3:**
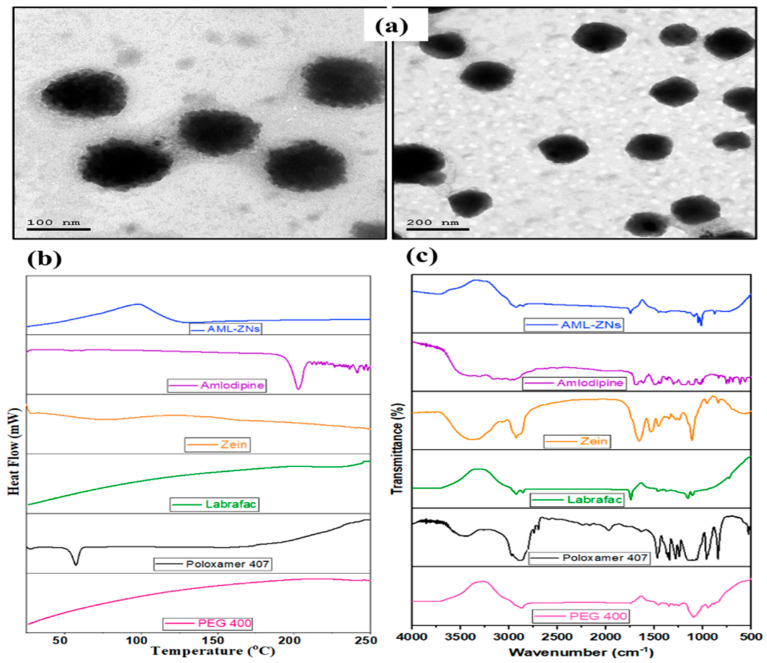
Further evaluation of the optimal AML-ZNs: (**a**) TEM image of zein polymeric nanoparticles; reflected spherical shape in monodisperse nano range, (**b**) Differential scanning calorimetry thermograms, and (**c**) Fourier transform infrared spectra for free amlodipine, Zein, Labrafac, poloxamer 407, PEG 400, and optimum AML-ZN formulation indicate optimal ZN fabrication and efficient drug loading.

The morphological evaluation of the optimized AML-ZNs conducted by TEM image, as shown in Figure 3a, revealed a regular size distribution with well-defined non-aggregated spherical particles, congruent with the image demonstrated by Araújo et al. [81]. The image reflected that the size of the optimum formulation was within the evaluated nanometric range, confirming the Zetasizer’s readings.

#### 3.2.2. Differential Scanning Calorimetry

DSC thermograms provide evidence about the thermal properties of an AML-ZN formulation, AML physicochemical state, and AML-additive compatibility in the formulation by detecting any alteration in endotherms and exotherms. As illustrated in Figure 3b, a pure AML thermogram displays an endothermic peak at about 210 °C. This result revealed the crystalline nature of AML and reflected its melting point, which agrees with previous findings of Bukhary et al. [82]. Conversely, the AML characteristic peak departure from the AML-ZN thermogram underlines AML solubilization and functional encapsulation of the drug amorphous phase into the zein matrix [23].

#### 3.2.3. Fourier Transform Infrared Spectroscopy

FT-IR established an applicable approach upon tracking AML characteristic peaks for affirming drug solubilization and efficient encapsulation into the ZN system. FT-IR spectra of amlodipine, zein, Labrafac, Poloxamer 407, PEG 400, and optimized AML-ZN formulation are illustrated in Figure 3c. FT-IR Spectra of pure AML presented different peaks, primarily at around 3037 cm^−1^ due to the hydroxyl group (OH), and a band assigned to the N-H group was shown at 3352 cm^−1^. A band ascribed to the stretching group of the C=O measured at about 1635 cm^−1^ [83]. The absence of amlodipine-specific peaks in AML-ZN spectra, as represented in Figure 3c, points out that the drug (AML) is no longer in its free crystalline form, instead implying incorporation into the nanoparticle matrix [84]. This disappearance indicated changes in the physical state and confirmed the fabrication of zein nanoparticles with competent drug encapsulation [22]. A previous investigation conducted by Mahmood et al. demonstrates that the disappearance of sharp peaks in the drug indicates that the drug is shifting to an amorphous state, which points to effective encapsulation [85]. Furthermore, Tulain et al. employed FT-IR spectra to prove the encapsulation of the drug into polymeric nanoparticles by tracking any shifting or disappearance of characteristic drug peaks, indicating solubility enhancement and confirming drug encapsulation [86].

### 3.3. Eyedrop Characterization

Due to the distinct structure of the eye and the necessity of specific properties regarding eye therapy, many features are in demand in eyedrop formulations to ensure applicability and bioavailability. Viscosity, pH, and sterility were essential properties to be considered upon preparation [27].

#### 3.3.1. Viscosity

Viscosity is crucial in ensuring an acceptable retention time of ophthalmic preparations to balance optimal contact time and avoid discomfort or blurred vision [87]. Viscosity enhancement to the 15–30 cps range elevates precorneal retention and improves drug delivery and bioavailability [88]. The optimized prepared AML-ZN formulation showed a measured viscosity value of 1.5 ± 0.07, considered a scant value that led to easy drainage by blinking or lacrimation [89]. Sodium alginate as a coating polymer was utilized to establish desirable features of improved viscosity and maintain stability [24]. Furthermore, as reported by Yao et al., coating zein nanoparticles with a polymer like alginate avoids hydrophobic attraction and nanoparticle aggregation and upholds system stability [90].

##### Adjust Eyedrop Viscosity

As represented in Table 4, 6 AML-ZNs-Alg formulations were prepared and evaluated to accomplish the optimum eyedrop viscosity. As noticed with the resulting values, increasing the alginate amount enhances the system’s viscosity. These outcomes are consistent with Figueroa-Enriquez et al.’s previous work, which found that viscosity increases with higher alginate concentrations [24]. Regarding the pseudoplastic behavior of alginate as a polysaccharide, the viscosity of the polymeric structure is prone to being controlled by the shear rate. Higher alginate concentration provokes the polymer chains’ entanglement, producing higher system viscosity [91]. Additionally, propylene glycol may enhance the polymer swelling process and slightly increase viscosity [92]. Only C3 and C6 acquired a viscosity value within the target range, with preference to C6 owing to the higher value. However, additional features should be evaluated for the AML-ZNs-Alg system to ensure the applicability of the alginate coating in this study.

##### Re-Evaluation of Critical AML-ZN Parameters

Dynamic light scattering

In Table 4, PS readings indicate an increase in the nanosize of the AML-ZN system upon coating with alginate polymeric nanoparticles as a response to additional layers settling on the surface of the AML-ZNs [21]. Moreover, higher augmentation of the Alg amount increases PS, as reported by Liu et al.’s previous study [20]. However, polymeric nanoparticles in the 50–400 nm range have been established as a versatile and efficient size in ophthalmic drug delivery [17]. Hence, the elevation in PS of Alg-coated AML-ZNs in C4, C5, and C6 formulations was applicable, as PS values were within the acceptable range, with preference to C6, and Alg coating amplifies the viscosity and stability attributes. Elevated concentration of PG as a plasticizer in the formulation from C4 to C6 produced a smaller particle size. As discussed before by Masalova et al., stabilizers in alginate nanoparticles can uphold small diameters by avoiding particle interactions and system collapse [93]. Also, Lee et al.’s previous study upholds PG as a nanoparticle stabilizer that prevents particle aggregation [94].

Regarding PDI, as represented in Table 4, the smallest alginate amount (50 mg) showed an increase in PDI values. This result is consistent with Takalani et al., as it may indicate insufficient alginate for optimum coating [95]. However, at the optimum alginate amount, the PDI showed a lower value in response to hardening due to the bond of the coating, resulting in a more uniform distribution [96]. Further increases in alginate may enhance PDI due to the high PDI values of Alg itself, referring to the variability in the range of polymerization and M.wt [97]. Moreover, higher alginate levels can induce uneven and faster cross-linking, resulting in elevated PDI [98]. The lower PDI value resulting from the higher PG% can be explained as increasing stabilizing agent concentration, enhancing viscosity, thus avoiding aggregation and upholding small particles with uniform distribution [99].

Considering ZP, alginate coating resulted in a highly negative surface charge compared to the positive charge of AML-ZNs. As represented in Table 4, all coating formulations acquired an elevated surface charge exceeding −30 mV. Higher absolute ZP over 30 mV ensures long-term system stability [75]. However, all AML-ZN formulations reflected a lower positive surface charge (<20 mV), which may affect stability. Honary and Zahir declared in previous work that formulations in the range of −20 mV to +20 mV are considered to have short-term stability [75]. A highly negative charge can be regarded as suitable for ocular therapy to ensure system stability, and it can support acceptable corneal penetration [100]. The negative sign accounted for appropriate corneal penetration for deep infection treatment [101].

Entrapment efficiency

As demonstrated in Table 4, all formulations post-coating revealed higher AML encapsulation than the optimized AML-ZNs, with increased entrapment percentage observed by the rise in alginate amount. The coating layer resulting from the deposition of alginate can enhance drug entrapment [21]. Additionally, elevated PG concentration as a cosolvent improves lipophilic AML’s solubility, which may aid the drug entrapment in the coating layer. As previously noted by Sharma et al., enhanced lipophilic drug solubility led to elevated drug–polymer interaction and miscibility, resulting in higher drug entrapment [102]. C6 is the selected AML-ZNs-Alg formulation for further investigation, as it acquired the optimum viscosity as an eyedrop preparation, with acceptable PS, PDI, and ZP alongside higher EE and pH. Otherwise, pH will undergo further adjustment for convenient ophthalmic delivery.

Vesicle morphology

**Figure 4:**
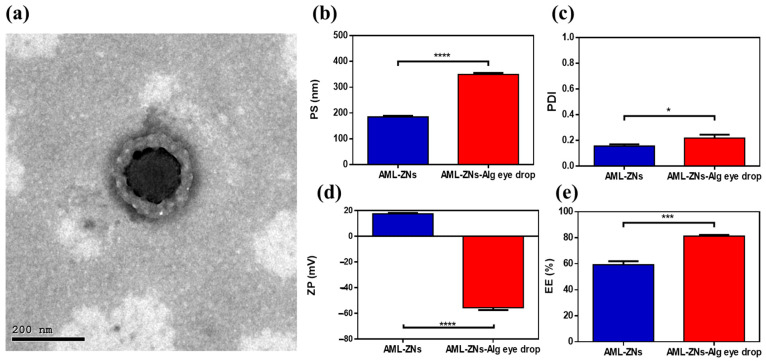
(**a**) TEM image of alginate-coated zein polymeric nanoparticles, representing spherical core-shell formation. Comparative studies of AML-ZNs versus AML-ZNs-Alg eyedrops considering (**b**) particle size, (**c**) polydispersity index, (**d**) zeta potential, and (**e**) entrapment efficiency. A significance level was developed at 0.05, and (* *p* < 0.05, *** *p* < 0.001, and **** *p* < 0.0001).

As depicted in Figure 4a, the morphological appearance of the selected AML-ZNs-Alg formulation was revealed with a spherical core-shell structure with a larger nanometric size, reflecting the alginate coating in the outer layer with a lighter shade than the core, as clarified previously by Tan et al.’s study [97,103].

#### 3.3.2. pH

Optimized AML-ZN formulations showed a pH value of 4.75 ± 0.14, while alginate coating formulations showed a slight increase, as displayed in Table 4. The slight pH enhancement can be explained by electrostatic forces of alginate deposited on the zein surface, which can mask the acidic moieties [104].

Adjusting the pH with phosphate buffer (pH: 7.4) before alginate coating causes system instability, as evidenced by the slight aggregation of ZNs. This may be consistent with Podaralla and Perumal’s previous observation that phosphate buffer may enhance zein nanoparticle aggregation, especially during lyophilization [60]. After coating, adjusting pH to 7.4 reflected no noticeable system instability, and all evaluated parameters showed insignificant changes in the mean value upon pH adjustment.

#### 3.3.3. Preservatives

PS, PDI, ZP, EE, and viscosity, after pH adjustment and 0.1% sodium benzoate incorporation, showed insignificant changes, as measured parameters were 349.9 ± 5.8 nm, 0.219 ± 0.0271, −55.45 ± 1.84, 81.29 ± 0.9%, and 19.3 ± 0.19 cp, respectively. This final preparation was selected for further comparative studies.

### 3.4. AML-ZNs and AML-ZNs-Alg Eyedrop Comparative Studies

#### 3.4.1. Dynamic Light Scattering Analysis and Entrapment Efficiency

Regarding the measurements of AML-ZNs-Alg eyedrops post optimization, as illustrated in Figure 4b, particle size assessment revealed a significant increase (*p* < 0.0001) in the PS of AML-ZNs after coating with alginate, which was previously clarified in Section 3.3.1. Coating improved stability and viscosity but resulted in size enlargement. However, the overall preference for coating encompasses the acceptable range of PS for ocular therapy, as it does not exceed 400 nm [105]. The comparative study regarding PDI, as represented in Figure 4c, revealed significant elevation (*p* < 0.05) post-coating with alginate. Nevertheless, a size distribution of less than 0.3 is considered applicable within the optimum limit [106]. Concerning zeta potential (Figure 4d), a significant shift (*p* < 0.0001) in surface charge from positive to highly negative post-coating occurred as alginate acquired negative charge [107]. Negatively charged alginate can uphold corneal penetration to treat deep infection, as clarified previously, and as advocated by ex vivo studies. The PS and ZP biodistribution curves for optimized AML-ZNs-Alg were clarified in Figure S2, which revealed a unimodal peak. Considering entrapment efficiency (Figure 4e), the outcome favored alginate coating, as the AML entrapment percent post-coating showed a significant increase (*p* < 0.001). Higher drug entrapment in polymeric nanoparticles can positively assist controlled drug release [108].

#### 3.4.2. In Vitro Release Study

**Figure 5:**
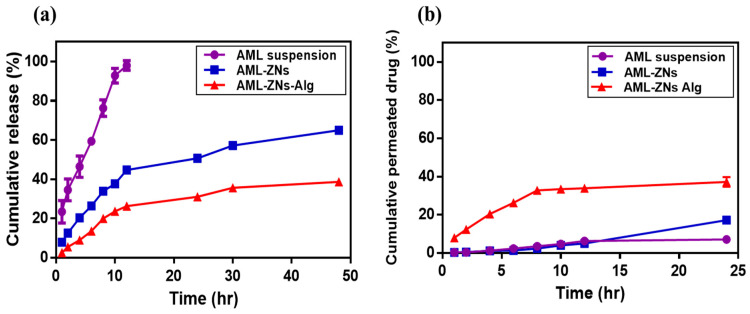
Comparative studies of (**a**) in vitro release and (**b**) ex vivo permeation demonstrate the cumulative proportion of amlodipine drug suspension compared to optimum AML-ZNs and AML-ZNs-Alg system.

Figure 5a shows the release patterns of optimized AML-ZNs and the selected AML-ZNs-Alg system compared to the AML suspension. As identified by the release profile, the AML suspension manifested a rapid release, with about 98% released after 12 h. On the contrary, AML-ZNs exhibited a biphasic release pattern: an initial burst release within the first 12 h, with about 45% released, followed by a gradual enhancement that reflected a more sustained manner as the total released AML increased by only about 20% after the following 36 h. The initial phase may be explained using the dissolution of free AML, which is unentrapped in the zein matrix [18]. Otherwise, as clarified in the previous study of Adel et al., the second phase of drug release from zein nanoparticles is controlled via the zein amphiphilic nature, as the hydrophobic regions restrict the entry of aqueous media. Moreover, the hydrophilic region facilitates the zein structure, enabling it to swell without eroding [67]. Concerning the AML-ZNs post-coating with alginate, the amount of drug released reached roughly 40% only after 48 h in a highly controlled manner. The alginate coating protected the zein nanoparticles’ surface, leading to a restricted diffusion with a slower release manner [97].

Applying various kinetics models to interpret the release profile suggested that the AML suspension release pattern was more relevant to the Korsmeyer–Peppas kinetics model, which was adopted as the best-fitting model, based on the higher R^2^ value of 0.9866. The value of the release exponent (n) was 0.632, revealing anomalous (non-Fickian) diffusion, which was controlled by AML’s lipophilic physicochemical features [45]. The Korsmeyer–Peppas model best described AML-ZNs and AML-ZNs-Alg release performances with an R^2^ of 0.9577 and R^2^ of 0.9267, respectively. The Korsmeyer–Peppas model can contribute to ascribing the drug release from a polymeric matrix [109]. Additionally, it is considered for systems where controlled release comprises multiple processes, not relying only on pure diffusion [110]. The exponent (n) values for AML-ZNs and AML-ZNs-Alg release were 0.450 and 0.489, respectively, indicating non-Fickian diffusion. As previously identified by Kang et al., the exponent values ranged from 0.45 to 0.9, which is related to a combined (non-Fickian) mechanism that considers diffusion and changes in polymer matrices [111].

#### 3.4.3. Ex Vivo Permeation and Deposition Studies

Ex vivo corneal permeation studies were conducted for AML suspension, optimized AML-ZNs, and the selected AML-ZNs-Alg system to ensure the penetration ability of formulated AML-ZNs and track the capability of negatively coating formulations in targeting deep corneal infection. As depicted in Figure 5b, the total percentage of AML permeated from the drug suspension post 24 h did not go beyond 7 ± 0.37%. Conversely, the total amount of AML permeated from ZNs and ZNs-Alg systems during the same period was about 17 ± 2.35% and 37 ± 3.52%, respectively. The permeation flux (Jss) values were 0.0047, 0.0092, and 0.0247 mg/cm^2^.h for AML suspension, AML-ZNs, and the AML-ZNs-Alg system. The lower permeation percentage of AML-ZNs precoating may be related to the observed aggregation on the corneal surface. As previously explained by various studies, the variation in repulsive forces, which are triggered by changes in physiological pH, temperature, and ionic strength, stimulates aggregation and instability features [61]. This attribute led to consideration of the zein nanoparticle as a weak colloid in terms of stability, favoring further coating to improve stability [112]. The permeation result post-coating proved that the anionic charge of alginate enhances the nanoparticle’s stability and ability to penetrate the corneal mucous layer deeply through repulsive forces [101]. In addition, the mucoadhesive feature of Alg can prolong precorneal residence time, enhancing AML absorption before precorneal loss [113]. Despite the charge, alginate mucoadhesive properties rely on physical entanglement and hydrogen bonds formed with the negatively charged mucin [114]. On the other hand, after carrying out the permeation study for up to 24 h, the estimated amount of deposited AML in the corneal strata was about 10.32 ± 1% for AML-ZNs compared to 8.5 ± 1.5% for the AML-ZNs-Alg system.

#### 3.4.4. Ex Vivo Visualization Study

**Figure 6:**
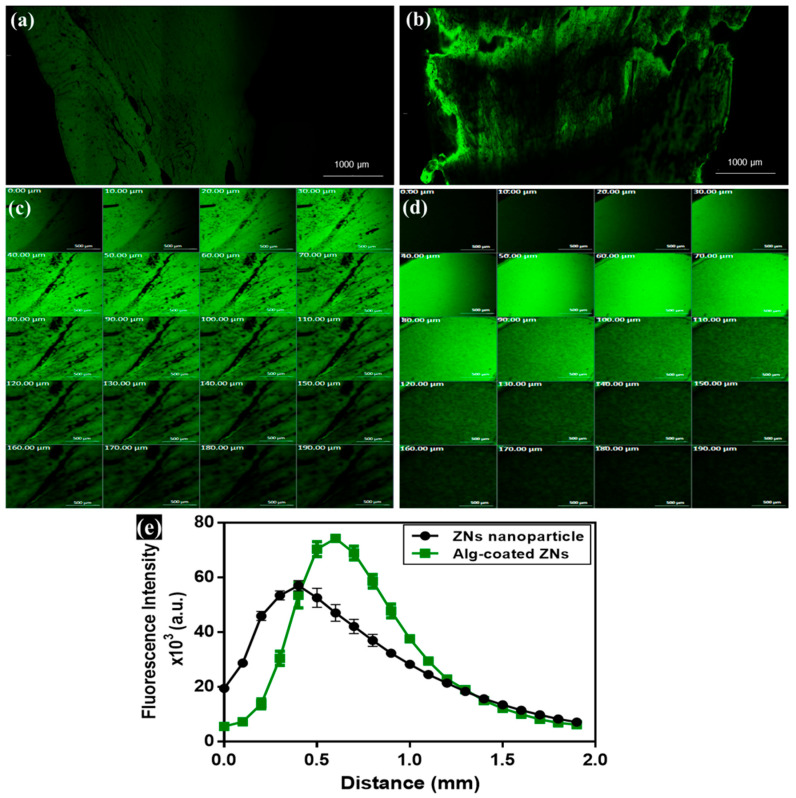
Confocal laser scanning microscopy images and tile x-y image of corneal tissue treated with (**a**) ZNs, (**b**) Alg-coated ZNs. Sequential images from 0–190 µm demonstrated penetration throughout several corneal layers: (**c**) ZNs, (**d**) Alg-coated ZNs, and (**e**) comparative quantitative assessment of fluorescence intensity distribution across corneal layers of ZNs and Alg-coated ZNs proved the preference of Alg-coated ZNs in penetration across deep corneal layers. Scale bar: 500 μm in (**b**,**c**).

Localization of ZNs and ZNs-Alg within corneal layers was assessed simultaneously by loading fluorescein diacetate (FDA) within formulations and tracking fluorescence intensity as a comparable study to prove permeability. CLSM images of Alg-coated ZNs represented higher penetration ability than ZNs, as indicated by the elevated dye intensity observed in the tile and sequential images, as shown in Figure 6a–d. Quantitative measurements of accumulated fluorescence in corneal layers for both formulations estimated preferable outcomes for the AML-ZNs-Alg system, reflected by an augmentation in depth and intensity, as depicted in Figure 6e. CLSM assessment validated permeation results and supported the capability of alginate coating to enhance nanoparticle penetration. These outcomes are consistent with those reported by Ger et al.; alginate acquired mucoadhesion properties that improve retention time on the corneal surface, upholding nanoparticle penetration and increasing the bioavailability of topical ocular therapy [26]. The ex vivo visualization investigation confirmed the localized retention and penetration of the fluorescently loaded nanoparticles in the ocular tissue. The tracked fluorescence intensity and distribution patterns indicated excellent mucoadhesion and surface contact, which are crucial for improving precorneal residence duration and ocular bioavailability, consistent with previous studies [114].

### 3.5. Storage Influence on AML-ZNs-Alg Eyedrops

**Figure 7:**
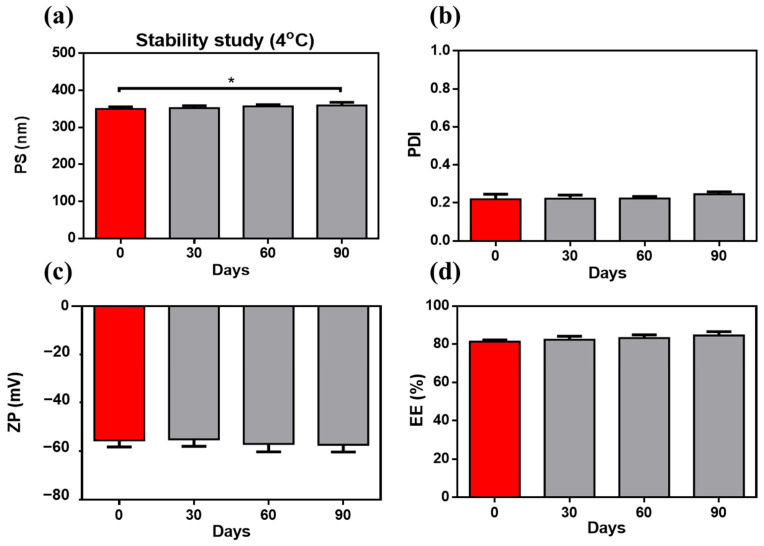
Stability study at 4 °C for 90 days: inspected sample, defined by (**a**) particle size, (**b**) polydispersity index, (**c**) zeta potential, and (**d**) entrapment efficiency; exhibits stable properties with only a slight increase in PS after 90 days in comparison to the fresh sample. A significance level was developed at 0.05, and (* *p* < 0.05 ).

Upon storage for 90 days, the ZNs-Alg system was revealed to preserve its yellowish colloidal appearance without noticeable phase separation or aggregation, as monitored by visual inspection. Compared to the fresh sample, pH and viscosity were maintained with insignificant changes (*p* > 0.05) upon storage at 4 °C. All assessment features, PS, PDI, ZP, and EE, exhibited insignificant alterations, as demonstrated in Figure 7a–d, except for a slight elevation in PS after 90 days. The increase in PS may be explained by the hydrophilic properties of alginate, which may absorb water and cause matrix swelling over time [115]. Alginate upholds zein stability and avoids system agglomeration by enhancing repulsive forces in response to the higher negative surface charge [22,24]. The tracking stability study affirmed the alginate coating’s ability to preserve system stability as desired.

### 3.6. In Vitro Antimicrobial Assessments

#### 3.6.1. Agar Well Diffusion Method

**Figure 8:**
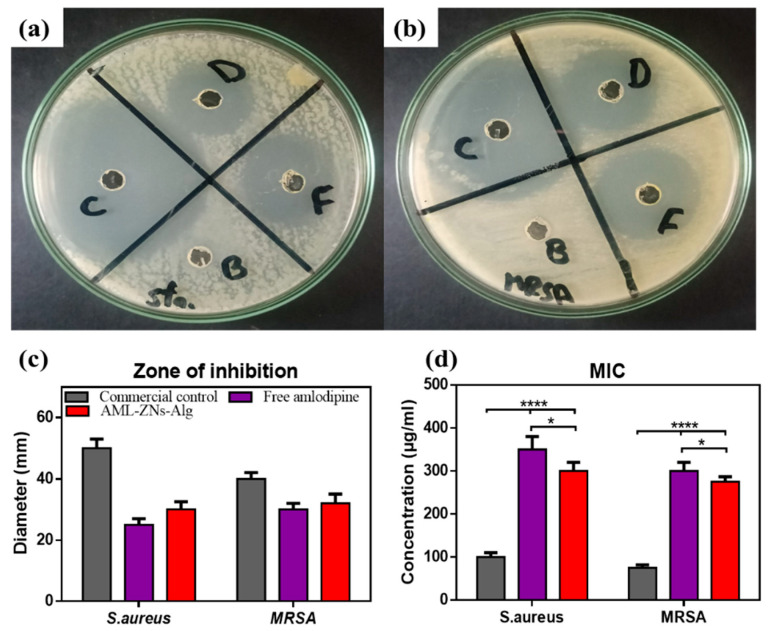
In vitro antimicrobial assessment: depictions of inhibition zones resulting from the activity of investigated formulations on (**a**) *Staph aureus* and (**b**) MRSA, with D, F, B, and C representing amlodipine-free drug, AML-ZNs-Alg formulation, Blank-ZNs-Alg, and commercial control, respectively. (**c**) Demonstrated results for inhibition zone diameters. The susceptibility of isolated bacterial species to free AML, AML-ZNs-Alg, and commercial control was estimated and statistically analyzed after evaluating (**d**) MIC values. The significance level was developed at 0.05, and (* *p* < 0.05, and **** *p* < 0.0001).

The growth inhibition zone is visualized and measured in Figure 8a–c for *S. aureus* and the MRSA strain. Outcomes concerning *S. aureus* show higher sensitivity to the AML-ZNs-Alg formulation (F) than to free AML, as the zone diameter was 30 mm. In comparison, the free AML (D) zone diameter was about 25 mm. In contrast, the Optiflox^®^ (commercial drug) (C) showed a higher value with a diameter of 50 mm (Figure 8a,c). On the other hand, the effect of the assessed formulations on the MRSA strain reflected slightly higher sensitivity to the AML drug, even pre- or post-formulation, than *S. aureus* (Figure 8b,c). The growth inhibition zone was induced to 30 mm by the free AML (D), while the AML-ZNs-Alg formulation (F) showed a slightly increased zone diameter of 32 mm. Meanwhile, regarding C, the inhibition zone value of 40 mm indicated less sensitivity of MRSA than *S. aureus* to the commercial drug. Blank-ZNs-Alg (B) showed no effect on both *S. aureus* and MRSA strains, as depicted in Figure 8a,b.

#### 3.6.2. Micro-Dilution Assay for MIC Determination

As illustrated in Figure 8d, MIC values for *S. aureus* and MRSA upon exposure to free AML, AML-ZNs-Alg, in addition to the commercial eyedrop (Optiflox^®^), were estimated and assessed statistically. MIC values proved the activity of AML as an antibacterial agent, while ZNs-Alg loaded with AML showed significantly lower MIC values than free AML (*p* < 0.05). The AML-ZNs-Alg system’s enhanced antibacterial efficacy, as reflected by the lower MIC values than those of the AML-free drug, can be explained by the controlled release mechanism resulting from drug incorporation into nanoparticles [116].

AML incorporation into a nanoparticle system established a suitable and powerful technique to enhance the efficacy of the drug as an antibacterial agent. Nanoparticles enhance drug stability, maintaining the controlled drug release, and stimulating penetration of antibacterial drugs across bacterial cell membranes and biofilm [117]. The previous study [118] summarized and concluded that antibiotics, focusing on gentamicin, post-incorporation into nanoparticles, promote the bactericidal effect, resulting in a wider inhibition zone diameter and a decrease in MIC values when measured against free drug [118]. The primary mechanism is unresolved, but the disruption of the biofilm and the adjustment of bacterial metabolism may be the basic causes, as they alter microbial sensitivity, making microbes more vulnerable to the drug [119].

Utilizing nanocarriers to enhance the antibacterial activity of the loaded drugs can include polymeric nanoparticles. Previous investigations mentioned that zein acts as a promising delivery carrier for antibiotics against several species, including *S. aureus*, upon enhancing drug performance and stability, and disrupting bacterial cells. Those studies noticed that zein alone is not considered an antibacterial remedy [120,121]. Furthermore, Jana and Jana previously discussed the synergistic antibacterial power of alginate as a carrier matrix in the case of loading antibacterial agents [122]. Thus, the in vitro study is consistent with previous findings, as AML-ZNs-Alg is preferred over free AML for antibacterial effect. At the same time, no inhibitory effect was detected with blank-ZNs-Alg.

The inclusive outcomes of in vitro antimicrobial assessments reinforce the efficacy of amlodipine as a promising remedy against bacterial infection, predominantly by resistant strains. Consistent with our results, as shown in previous considerations by Barbosa et al., AML activity was proven as an antibacterial therapy against *S. aureus* species, pointing to the drug’s synergistic effect with traditional antibiotics. The antibacterial mechanism of AML relies on bacterial membrane destabilization and cellular demise [123]. Regarding resistant strains such as MRSA, previous in vitro evaluation revealed the ability of AML to suppress various β-lactamases, particularly when co-administered with other antibiotics [124].

### 3.7. In Vivo Assessments

#### 3.7.1. Evaluation of Ocular Irritation

**Figure 9:**
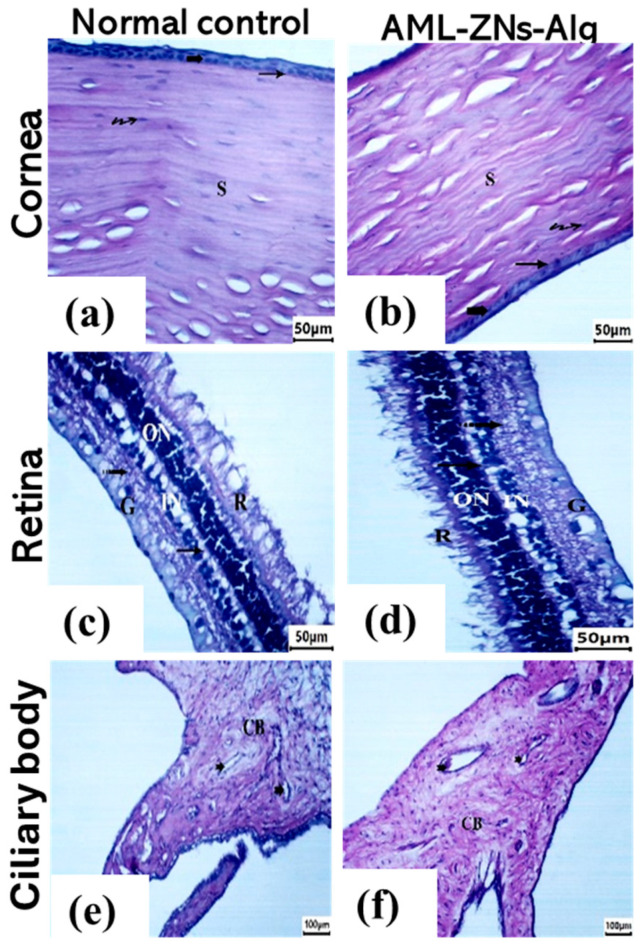
Ocular Draize test: corneal histological images for (**a**) normal control and (**b**) AML-ZNs-Alg, Retina histological images for (**c**) normal control and (**d**) AML-ZNs-Alg, and ciliary body histological images for (**e**) normal control and (**f**) AML-ZNs-Alg, showing normal histological features ensuring ocular biocompatibility of the prepared formulation. Symbols in corneal images indicate epithelium (bold arrow), Bowman’s membrane (arrow), stroma (S), and keratocytes (wavy arrow), while in retinal and ciliary bodies referred to photoreceptor layer (R), outer nuclear layer (ON), outer plexiform layer (arrow), inner nuclear layer (IN), inner plexiform layer (dotted arrow), ganglion cell layer (G), blood vessels (notch arrow), and ciliary body (CB).

The cornea, retina, and ciliary body were examined for the rabbit eye loaded with AML-ZNs-Alg compared to the normal control. Microscopic images of corneal tissue sections for the prepared formulation (Figure 9b) revealed a structure similar to that of normal control (Figure 9a), reflecting stratified squamous non-keratinized epithelium (bold arrow) with well-organized cells. An intact Bowman’s membrane (arrow) was detected underneath the epithelium. The stroma (S) appeared with regularly arranged collagen bundles, with flattened and uniformly distributed keratocytes (wavy arrow) between the stromal fibers. Retinal histological photomicrographs of AML-ZNs-Alg (Figure 9d) provided a comparable image of normal tissue (Figure 9c), demonstrating intact retinal pigmented epithelium and photoreceptor layer (R). All layers were structured and unaltered, encompassing the outer nuclear layer (ON), outer plexiform layer (arrow), inner nuclear layer (IN), inner plexiform layer (dotted arrow), and ganglion cell layer (G). Moreover, ciliary body histological images of normal control and AML-ZNs-Alg (Figure 9e,f) revealed identical features of normal blood vessels (notch arrow) of the ciliary body (CB), without any signs of congestion or inflammatory infiltration.

#### 3.7.2. In Vivo Antimicrobial Evaluation

**Figure 10:**
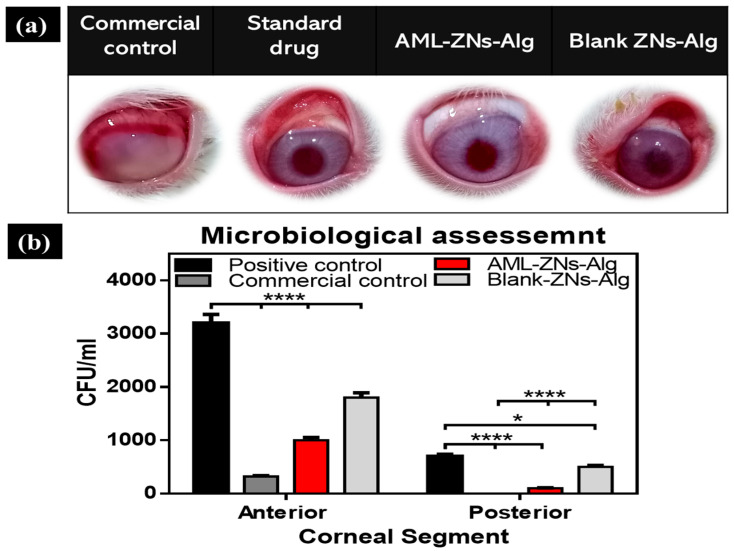
(**a**) Illustrative images of rabbits’ eyes 7 days post-treatment for the investigated groups comprising positive control, commercial control (Optiflox^®^), AML-ZNs-Alg eyedrops, and blank-ZNs-Alg. (**b**) In vivo antimicrobial evaluation employing bacterial ocular infection rabbit model: MRSA counts for corneal tissue in CFU/mL 7 days post-treatment for all investigated groups were determined. The significance level was developed at 0.05, and (* *p* < 0.05, and **** *p* < 0.0001).

Infected ocular tissue from rabbits was employed using the MRSA strain to induce infection and assess the activity of the prepared AML-ZNs-Alg and blank-ZNs-Alg eyedrop systems in comparison with the commercial control (Optiflox^®^) and positive control 7 days post-treatment, as shown in Figure 10a. After the treatment period, the corneal images of the studied groups showed severe infection affecting corneal and conjunctival tissues with intense ocular redness and ulceration. However, the commercial drug groups and AML-ZNs-Alg displayed a remarkable improvement in ocular appearance, with diminished redness and inflammation.

Additionally, microbial counts were quantified to prove efficacy and substantiate visual outcomes. Figure 10b demonstrates microbial counts after 7 days of treatment for both the anterior and posterior corneal segments. Regarding the anterior segment, the AML-ZNs-Alg system showed a microbial count of 1 × 10^3^ ± 0.1 × 10^3^ CFU/mL, compared to 3.2 × 10^2^ ± 0.16 × 10^2^ CFU/mL in the commercial drug group (*p* < 0.0001). Otherwise, the positive control count of 3.2 × 10^3^ ± 0.2 × 10^3^ CFU/mL, compared to that of the AML-ZNs-Alg system and the commercial drug, reflected a significantly higher bacterial burden (*p* < 0.0001). The microbial count for the blank-ZNs-Alg system was 1.8 × 10^3^ ± 0.19 × 10^3^ CFU/mL, a significantly higher bacterial load than those of the ZNs-Alg system and commercial drug (*p* < 0.0001). Concerning the posterior segment, the AML-ZNs-Alg system showed a scant microbial load of 1 × 10^2^ ± 0.05 × 10^2^ CFU/mL, insignificantly higher than the commercial drug group with no detectable load. The AML-ZNs-Alg system and the commercial drug, compared to the positive control with a count of 7 × 10^2^ ± 0.36 × 10^2^ CFU/mL, indicated a significant inhibition (*p* < 0.0001) in the bacterial count. The microbial count with the blank-ZNs-Alg system was 5 × 10^2^ ± 0.24 × 10^2^ CFU/mL, with a significantly elevated bacterial load compared to the results for the ZNs-Alg system and commercial drug (*p* < 0.0001).

The in vivo microbiological assessment outcomes highlighted AML’s activity as an antibacterial agent against MRSA, and supported the microbial testing in vitro. Previous investigations by Andrade et al. emphasized AML’s antibacterial efficacy against S. aureus, utilizing an in vivo evaluation of the Galleria mellonella model and proposing the drug as an emerging therapeutic remedy [125]. The blank-ZNs-Alg system was observed to show slight antibacterial activity in vivo. As discussed before, zein and sodium alginate-based nanosystems do not reveal apparent antibacterial activity in vitro without being coupled with bioactive agents [30,126]. However, intricate psychological environments can alter biopolymers’ features and induce their bioactivity during in vivo evaluations [127].

#### 3.7.3. Histopathological Dissection

**Figure 11:**
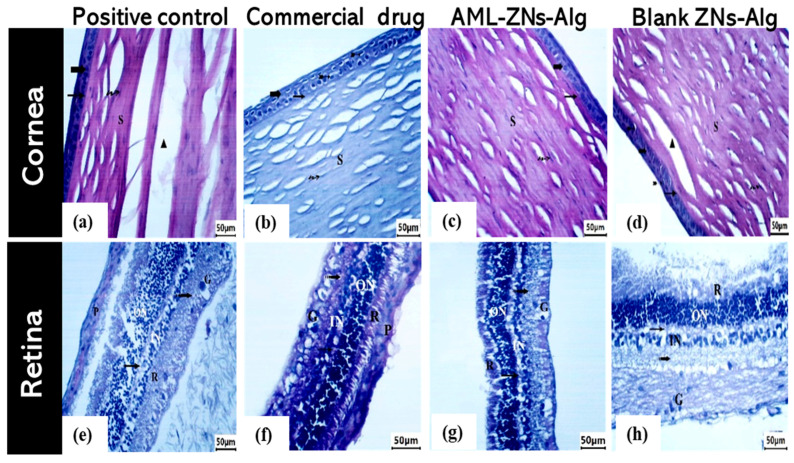
Corneal histopathological images for (**a**) positive control, (**b**) commercial control, (**c**) AML-ZNs-Alg, and (**d**) blank-ZNs-Alg. Retina histopathological images for (**e**) positive control, (**f**) commercial drug, (**g**) AML-ZNs-Alg, and (**h**) blank-ZNs-Alg. Symbols in corneal images indicate pyknotic nuclei (bifid arrow), stroma (S), large gaps (▲), epithelium (bold arrow), Bowman’s membrane (arrow), and keratocytes (wavy arrow), while in retinal images, symbols refer to the photoreceptor layer (R), outer nuclear layer (ON), outer plexiform layer (arrow), inner nuclear layer (IN), inner plexiform layer (dotted arrow), and ganglion cell layer (G).

The cornea, retina, ciliary body, and iris were assessed in rabbit eyes for all groups under investigation, which comprised AML-ZNs-Alg and blank-ZNs-Alg compared to the commercial drug (Optiflox^®^) and the positive control. A microscopic image of a cornea specimen from the positive control (Figure 11a) revealed deeply stained pyknotic nuclei (bifid arrow) in epithelial cells with detachment of the basement membrane (**) beneath the epithelium. Stromal collagen fibers (S) appear separated by large gaps (▲), pointing to degeneration. The Optiflox^®^ group, as shown in Figure 11b, seemed to have a nearly normal structure with an intact epithelium (bold arrow) and Bowman’s membrane (arrow). Deeply stained pyknotic nuclei cells (bifid arrow) existed. Stromal collagen fibers (S) were arranged but revealed slight spacing with organized flattened keratocytes (wavy arrow). The AML-ZNs-Alg histological corneal image (Figure 11c) resembled the commercial drug results. The corneal epithelium (bold arrow) and Bowman’s membrane (arrow) were completely intact. Regular, well-structured collagenous fibers were arranged in the stroma (S) with regular distribution of keratocytes (wavy arrow). The blank-ZNs-Alg specimen demonstrated, in Figure 11d, a mostly normal epithelium (bold arrow) with the existence of pyknotic nuclei cells (bifid arrow), while stromal collagen fibers (S) showed gap separation (▲).

Moreover, retinal histological photomicrographs for the commercial drug and AML-ZNs-Alg groups (Figure 11f,g) manifested an undamaged, well-structured appearance for all layers encompassing retinal pigmented epithelium (RPE), photoreceptors, outer and inner nuclear and plexiform layers, and ganglion cell layer. On the contrary, the positive control and blank-ZNs-Alg group showed enhanced retinal layer thickness, as depicted in Figure 11e,h.

**Figure 12:**
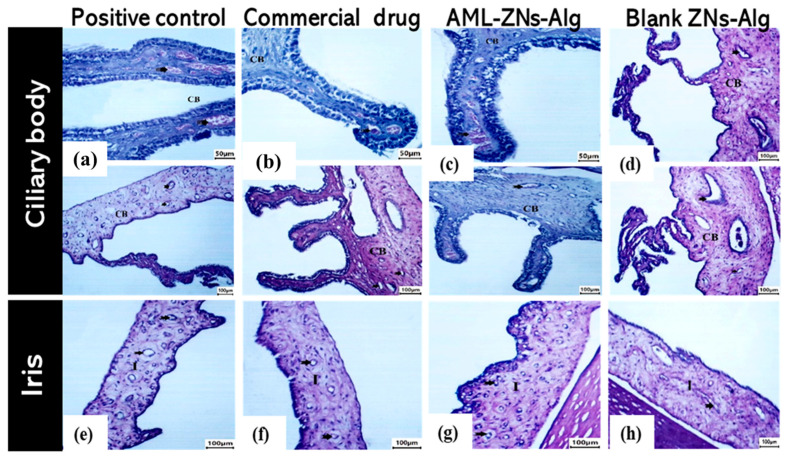
Ciliary body histopathological images for (**a**) positive control, (**b**) commercial control, (**c**) AML-ZNs-Alg, and (**d**) blank-ZNs-Alg. Iris histopathological images for (**e**) positive control, (**f**) commercial drug, (**g**) AML-ZNs-Alg, and (**h**) blank-ZNs-Alg. Symbols were used to indicate blood vessels (notch arrow), ciliary body (CB), iris (I), and congestion (bold arrow).

Additionally, ciliary body and iris histological images from the positive control group showed vascular congestion in the iris and ciliary body (bold arrow) (Figure 12a,e). The commercial drug (Figure 12b,f) and AML-ZNs-Alg (Figure 12c,g) groups revealed preserved normal blood vessels (notch arrow) of the ciliary body (CB) and iris (I), with detection of mild congestion in the commercial drug group (bold arrow).

## 4. Conclusions

The current investigation efficiently established an inventive therapeutic system for delivering amlodipine besylate (AML), topically targeting ocular tissues as a repositioning cure for bacterial infection. The zein nanoparticles (ZNs) were demonstrated to be a convenient and efficient carrier for AML corneal administration, resulting in outstanding outputs. The optimized composition of zein nanoparticles consisted of 2.068% (*w*/*v*) zein, 0.75% (*w*/*v*) Labrafac, and 1% (*w*/*v*) Poloxamer. The optimized formulation (AML-ZNs) was coated with the selected concentration of alginate polymer to adjust system stability and viscosity. Eyedrop features, considering viscosity, pH, and sterility, were maintained to enhance system convenience. The comparative studies of AML-ZNs-Alg compared to AML-ZNs confirm the suitability of coating with a preference for stability, drug loading, controlled release, and corneal penetration. The alginate coating of AML-ZNs produced a more controlled release pattern, as only 40% of AML was released from the ZNs-Alg after 48 h compared to 65% from ZNs. In addition, the coating system enhanced drug penetration via corneal tissues, as upheld by CLSM visualization assessment outcomes. Tracking AML-ZNs-Alg eyedrop features during storage at refrigerator temperature for up to 90 days assured system stability and observed insignificant manifestation. The diminishing microbial count with the AML-ZNs-Alg formulation in the in vitro and in vivo antimicrobial evaluations demonstrated the efficacy of AML as a repositioning remedy with promising potential activity against MRSA strains. Moreover, histological pathology findings established evidence of AML activity and tracking formulation safety. However, future investigations encompassing a broad sample size for in vivo assessment associated with long-term safety studies are desired. Additionally, studying the synergistic effects of AML combined with other recognized antibiotics is needed to provide a wide therapeutic framework for targeting better therapeutic outcomes for ocular infection. Regardless of the promising outcomes concerning preclinical evaluation, translating these findings into clinical application faces various obstacles regarding dose optimization, maintaining the drug’s therapeutic index, and assuring systemic safety, which need to be considered in future studies.

## Figures and Tables

**Table 1:** Manipulated variables and measured outcomes employing a Box–Behnken design to optimize AML-ZNs.

Manipulated Variables	Levels	
	(−1)	(1)
X_1_: Zein concentration (%)	1.5	2.5
X_2_: Labrafac concentration (%)	0.75	2.25
X_3_: Poloxamer 407 concentration (%)	0.5	1
Measured outcomes	Desirability constraints	
Y_1_: Particle size (nm)	Minimize	
Y_2_: Polydispersity index	Minimize	
Y_3_: Zeta potential (absolute value) (mV)	Maximize	
Y_4_: Entrapment efficiency (%)	Maximize	

**Table 2:** AML-ZN formulations according to the Box–Behnken design (BBD).

	Manipulated Variables			Measured Outcomes			
F#	Zein Concentration (X_1_)	Labrafac Concentration (X_2_)	Polaxemer 407 Concentration (X_3_)	Particle Size (nm) Y_1_	Polydispersity Index Y_2_	Zeta Potential (mV) Y_3_	Entrapment Efficiency (%) Y_4_
Z1	2.5	1.5	1	221.9 ± 5.11	0.200 ± 0.0201	15.25 ± 0.36	65.33 ± 1.27
Z2	2	0.75	0.5	211.2 ± 4.56	0.199 ± 0.0079	16.64 ± 0.23	56.53 ± 3.83
Z3	2	1.5	0.75	204.0 ± 3.85	0.233 ± 0.0017	15.89 ± 0.19	54.33 ± 2.72
Z4	2.5	2.25	0.75	234.4 ± 1.72	0.249 ± 0.0024	15.93 ± 0.29	73.38 ± 2.67
Z5	1.5	0.75	0.75	173.7 ± 9.69	0.145 ± 0.0053	18.87 ± 0.14	39.60 ± 4.98
Z6	2	0.75	1	186.0 ± 10.03	0.161 ± 0.009	18.35 ± 0.22	60.44 ± 3.02
Z7	1.5	1.5	1	182.8 ± 8.14	0.240 ± 0.0021	17.05 ± 0.15	36.42 ± 3.82
Z8	2	2.25	1	207.1 ± 6.36	0.272 ± 0.0106	15.19 ± 0.56	51.47 ± 2.57
Z9	2	1.5	0.75	223.4 ± 4.17	0.201 ± 0.0111	17.39 ± 0.21	58.02 ± 0.90
Z10	1.5	2.25	0.75	207.6 ± 7.38	0.284 ± 0.0142	15.65 ± 0.28	33.04 ± 1.65
Z11	2.5	1.5	0.5	237.4 ± 3.87	0.254 ± 0.0127	16.51 ± 0.33	74.04 ± 1.70
Z12	2	1.5	0.75	219.3 ± 6.97	0.231 ± 0.0115	17.42 ± 0.57	63.81 ± 3.19
Z13	2	1.5	0.75	203.4 ± 8.17	0.195 ± 0.0098	18.51 ± 0.13	54.24 ± 2.71
Z14	2	1.5	0.75	204.4 ± 7.22	0.207 ± 0.0004	17.58 ± 0.23	57.62 ± 2.88
Z15	1.5	1.5	0.5	200.3 ± 10.02	0.202 ± 0.0101	17.39 ± 0.17	38.50 ± 1.93
Z16	2.5	0.75	0.75	226.2 ± 5.31	0.212 ± 0.0106	15.81 ± 0.09	71.09 ± 1.55
Z17	2	2.25	0.5	233.8 ± 11.69	0.251 ± 0.0125	16.74 ± 0.14	56.07 ± 2.80

**Table 3:** ANOVA results for BBD responses of AML-ZN formulations.

Response	R^2^	Adjusted R^2^	Predicted R^2^	Adequate Precision	Model	F-Value	*p*-Value	Significance
PS (nm)	0.8914	0.8663	0.8390	18.4560	Linear	35.56	<0.0001	Significant
PDI	0.9273	0.8838	0.8846	16.7791	2FI	21.27	<0.0001	Significant
ZP (mV)	0.7085	0.5336	0.4659	8.2082	2FI	4.05	0.0254	Significant
EE (%)	0.9304	0.9143	0.8855	20.9620	Linear	57.89	<0.0001	Significant

Abbreviations: PS, particle size; PDI, polydispersity index; ZP, zeta potential; EE, entrapment efficiency.

**Table 4:** AML-ZNs-Alg formulation variables and evaluated parameters.

	Variables		Evaluated Parameters					
F	Alginate (mg)	PG % (*w*/*v*)	Visc. (cp)	PS (nm)	PDI	ZP (mV)	EE (%)	pH
C1	50	2	4.61 ± 0.27	416.2 ± 4.53	0.403 ± 0.0232	−57.51 ± 1.25	68.32 ± 2.31	4.89 ± 0.02
C2	100	2	7.64 ± 0.21	438.9 ± 7.31	0.276 ± 0.0573	−58.62 ± 2.56	72.15 ± 1.82	4.97 ± 0.01
C3	150	2	15.22 ± 0.25	454.6 ± 2.36	0.327 ± 0.1011	−65.06 ± 1.13	77.96 ± 1.72	5.13 ± 0.02
C4	50	4	6.13 ± 0.35	312.1 ± 10.97	0.385 ± 0.0421	−53.72 ± 2.44	72.57 ± 2.13	5.11 ± 0.03
C5	100	4	13.15 ± 0.17	330.3 ± 7.52	0.194 ± 0.0351	−54.72 ± 1.18	79.47 ± 1.62	5.25 ± 0.02
C6	150	4	19.05 ± 0.17	349.3 ± 4.61	0.210 ± 0.0305	−56.91 ± 3.45	83.19 ± 1.91	5.38 ± 0.03

Abbreviations: PG, propylene glycol; Visc., viscosity; PS, particle size; PDI, polydispersity index; ZP, zeta potential; EE, entrapment efficiency.

## Data Availability

The data presented in this study are available on request from the corresponding author.

## Supplementary Materials

The following supporting information can be downloaded at: https://www.mdpi.com/article/10.3390/pharmaceutics17101314/s1, Figure S1: (a) AML-ZNs particle size distribution curve shows a unimodal peak centered around 185 nm, indicating a uniform nanoparticle population, and (b) AML-ZNs zeta potential distribution curve with a peak around 18 mV; Figure S2: (a) AML-ZNs-Alg particle size distribution curve shows a unimodal peak centered around 350 nm, indicating a uniform nanoparticle population, and (b) AML-ZNs-Alg zeta potential distribution curve with a peak around −55 mV, reflecting good electrostatic stability.
