# Supplementary material for: Coated Zein Polymeric Nanoparticles Loaded with Amlodipine as a Repurposed Antibacterial Ocular Cure for MRSA-Induced Infection: Optimization, In Vitro, Ex Vivo, and In Vivo Assessments
Source: Pharmaceutics. 2025 Oct 10;17(10):1314. doi: 10.3390/pharmaceutics17101314 (PMC12566698; doi:10.3390/pharmaceutics17101314)
Supplement: Supplementary file 1 [file pharmaceutics-17-01314-s001.zip › pharmaceutics-3902451-supplementary.pdf]

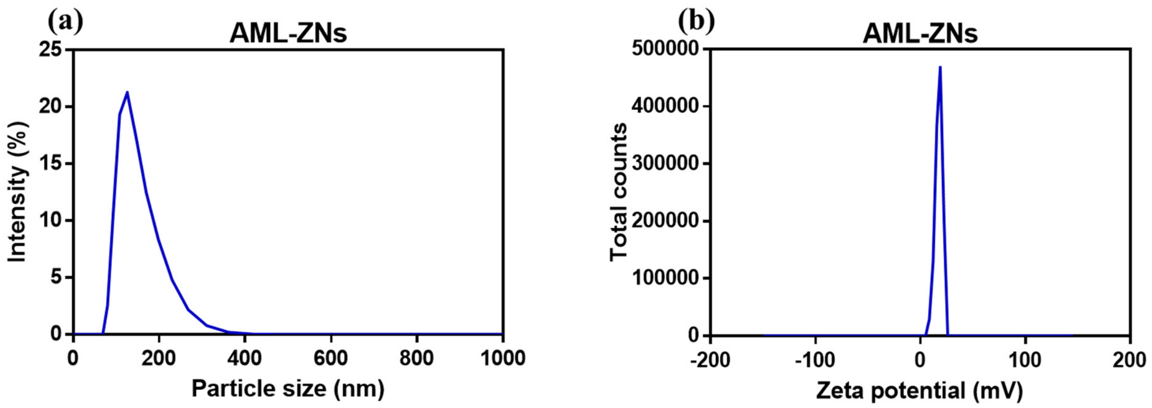


**Figure S1.** (a) AML-ZNs particle size distribution curve shows a unimodal peak centered around 185 nm, indicating a uniform nanoparticle population, and (b) AML-ZNs zeta potential distribution curve with a peak around 18 mV.

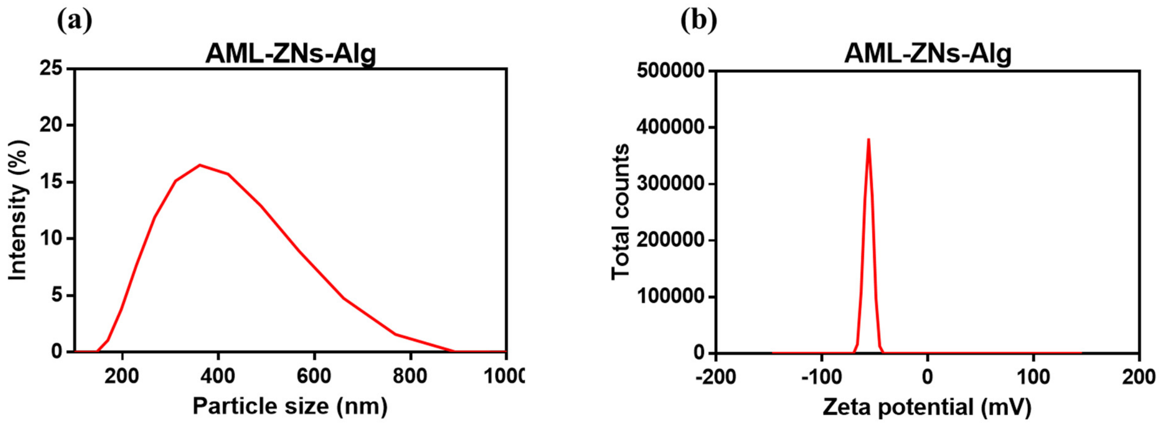


**Figure S2.** (a) AML-ZNs-Alg particle size distribution curve shows a unimodal peak centered around 350 nm, indicating a uniform nanoparticle population, and (b) AML-ZNs-Alg zeta potential distribution curve with a peak around -55 mV, reflecting good electrostatic stability.
